# Interaction of Smoking and Genetic Polymorphisms in the Dopaminergic Pathway in Relation to Parkinson’s Disease: A Scoping Review

**DOI:** 10.7759/cureus.90055

**Published:** 2025-08-14

**Authors:** Nida Cansu Yavuz, Khushi Nayyar, Francesca Pappafava, Jishnu Praveen, Anandita N Nair, Mert Umut Bora, Fawaz Aldoohan, Abdur Rahmaan Mohammed Yasin Hudli, Yagnik Reddy Siddenki, Sadia Nazir, Hossam Tharwat Ali

**Affiliations:** 1 Faculty of Medicine, Dokuz Eylül University, İzmir, TUR; 2 Department of Internal Medicine, Adesh Institute of Medical Sciences & Research, Bathinda, IND; 3 Faculty of Medicine and Surgery, Università degli Studi di Perugia, Perugia, ITA; 4 Faculty of Medicine, Stanley Medical College, Chennai, IND; 5 Department of Medicine, Our Lady of Fatima University, Valenzuela, PHL; 6 Department of Emergency Medicine, Iğdır Dr. Nevruz Erez Devlet Hastanesi, Iğdır, TUR; 7 Department of Family Medicine, American Academy of Research and Academics, New Castle, USA; 8 Faculty of Medicine, Our Lady of Fatima University, Valenzuela, PHL; 9 Department of Medicine, Prathima Institute of Medical Sciences, Karimnagar, IND; 10 Department of Medicine, Shaheed Mohtarma Benazir Bhutto Medical College Lyari, Karachi, PAK; 11 Qena Faculty of Medicine, South Valley University, Qena, EGY

**Keywords:** comt, dopamine, monoamine oxidase, parkinsonism, parkinson’s disease, pd, polymorphism

## Abstract

The inverse association of Parkinson’s disease (PD) and smoking has been well established, but it is rarely investigated in the context of the different genetic backgrounds of the patients. This scoping review aims to comprehensively review the literature on the interactions of different essential genes involved in the dopaminergic pathway with smoking and how they modulate the risk of PD with smoking. A search using the keywords and abbreviations of smoking, genetic polymorphism, and parkinsonism was conducted in Web of Science, PubMed, Cochrane, and Scopus databases. References were retrieved and screened by two authors independently. Eleven human studies were included with 5131 cases and 7542 control adult populations with a male predominance (>60%) and multiethnic groups, mainly European origin populations. Most gene polymorphisms showed no modulation of the smoking-PD risk. Nevertheless, the risk was shown to change with MAO-B rs1799836 gene polymorphism in three studies where the G allele was associated with smoking protection against PD in 16.5% and 13.14% of cases and controls, respectively. While the COMT rs4680 polymorphism was suggested to interact with smoking in one study with 238 (37.9%) cases and 369 (31.5%) controls (p-value = 0.061), the effect of smoking on PD risk varied significantly with different SV2C (rs30196 and rs10214163) genotypes. Our study revealed the complexity of genetic polymorphism in the smoking-PD relationship. This explores the possibility of further research with larger sample sizes, accounting for different ethnic groups, to establish definitive links that can aid in treatment and prevention strategies.

## Introduction and background

Parkinson’s disease (PD), a progressive neurodegenerative disease, affects around 2% of people aged above 60 and 4% of people aged over 80, with a prevalence increasing with age [1,2]. The characteristic symptoms of PD include resting tremors, rigidity, loss of postural reflexes, and bradykinesia [1-3]. PD, a significant cause of morbidity, is associated with healthcare-related costs and burdens [4]. Furthermore, patients with PD and their caregivers sustain a significant economic burden due to hospitalizations, medications, and indirect costs [5]. Therefore, determining sociodemographic, genetic, and environmental factors that can predispose to disease development or aid in disease prevention and outcome remains imperative. The aforementioned goal can be achieved by customized treatments for PD, which can be guided by determining individual risk factors [6].

A decrease in the dopaminergic neurons, hence dopamine, in the substantia nigra pars compacta and α-synuclein aggregation were shown in PD as the predominant pathologic mechanism [1,6,7]. Pathological examination of PD patients shows a common feature of dopaminergic neuron loss [8]. This loss results in the significant denervation of the nigrostriatal pathway. Although the definitive mechanism behind pathophysiology remains unclear, the outcome, prognosis, and risk of PD have been shown to have associations with particular environmental factors, including smoking, caffeine, and high serum urate [9].

One of the factors that also has a prominent relationship with PD is genetics. Most PD cases are genetically influenced, with at least 100 genetic loci identified [10]. While several genes have been investigated in the context of PD, some are involved in common functional pathways. For instance, many genes involved in the dopaminergic pathway have been mentioned, including genes encoding dopamine receptor D (DRD), monoamine oxidase A and B (MAO-A/B), and catechol-o-methyltransferase (COMT) [1,11]. Being involved in dopamine metabolism, MAO-B, one of the metabolizers of dopamine, can contribute to PD by activating a toxic metabolite, 1-methyl-4-phenylpyridinium (MPP+) [12]. Similarly, COMT deactivates the dopamine precursor [13]. Other genes were mentioned to be involved in the dopamine release mechanism, such as synaptic vesicle glycoprotein 2C (SV2C) [14].

Specifically, an inverse relationship with smoking was shown, prompting the question of whether smoking has a neuroprotective effect against PD [15,16]. Particular animal studies suggested that the reduced risk associated with smoking can be due to the dopaminergic effect of smoking [17]. Nicotine, a key component of cigarette smoke, stimulates nicotinic acetylcholine receptors that promote dopaminergic signaling and hence motor function. Certain chemicals in cigarettes can inhibit the MAO enzymatic activity [15-17]. Additionally, smoking has different effects on gut microbiome, motility, and pro- and anti-inflammatory effects; however, whether these alterations play a role in the reduced PD risk remains unknown [18]. The molecular basis of this inverse relationship is important to understand for the future of PD treatment and stopping disease progression [19].

Faced with complexity, investigators have recognized the need to study epigenetic mechanisms of the interactions between genes and environmental factors [20,21]. The association of either smoking or genetic variants with PD separately has been classically studied, with only a minority of studies investigating the possible gene-smoking interaction in the context of PD [13,22-29]. However, this scoping review is the first comprehensive review that examines the possible interactions between smoking and genetic polymorphism on PD. Herein, we aimed to provide a thorough evaluation of the current evidence on the interaction of smoking and particular genes involved in the dopaminergic pathway in the context of PD. The present article aims to conclude whether genetic polymorphism of genes of dopaminergic pathways modulates the established effect of smoking on PD.

## Review

Methods

Review Design and Question

This study was conducted as a scoping review, which is a method that aims to search and synthesize the existing data on a particular topic using a systematic approach [30]. It remains unclear whether interactions between the genes involved in the dopaminergic pathway and smoking affect disease progression, risk, or other possible aspects of PD [31]. We aimed to provide a thorough look at the question of whether an interaction between the genetic variants or polymorphisms involved in the dopaminergic pathway with smoking affects the risk of PD in any way. Our question has been raised due to the need for a comprehensive look at the topic. Therefore, a scoping review approach was selected [32]. The Preferred Reporting Items for Systematic reviews and Meta-Analyses extension for Scoping Reviews (PRISMA-ScR) was used for guidance [33].

Literature Search and Eligibility Criteria

A search using the keywords and abbreviations of smoking, genetic polymorphism, and parkinsonism was conducted in Web of Science, PubMed, Cochrane, and Scopus databases on September 22, 2024. Inclusion criteria consisted of human observational studies in the English language only. Case reports, reviews, animal studies, in vitro studies, articles that mention no interactions with smoking, articles that do not mention any genetic element or polymorphisms, articles that were irrelevant to PD, and articles that could not be retrieved were strictly excluded (Table 1).

**Table 1:** Summary of the scoping review methodology

Searched databases	
Web of Science, Scopus, PubMed, and Cochrane Library	
Search strategy	
(“Smoking” OR “tobacco” OR “smoke” OR “smoker” OR “smokers” OR “nicotine” OR “nicotinic” OR “cigarette”) AND (“Parkinson” OR “Parkinson’s” OR “Parkinsonism”) AND (“Gene” OR “Genetic” OR “variant” OR “polymorphism” OR “genome” OR “allele” OR “mutant” OR “mutation” OR “Genic” OR “polygenic”)	
Inclusion criteria	Exclusion criteria
Human studies, published in the English language, with a retrievable full text, investigate the interaction of genes involved in dopamine pathways and smoking on PD risks	Animal studies, in vitro studies, case reports, reviews, or articles in a non-English language

Selection of the Studies

The results obtained from the search were recorded on a Microsoft Excel sheet (Microsoft Corporation, Redmond, WA, USA) for convenience. Each article was thoroughly screened by two authors independently, and the decision of inclusion or exclusion was made according to the aforementioned criteria and the relevance to our research question. To minimize bias, a third independent author reviewed the included articles when conflicts occurred.

Data Extraction and Analysis

The final articles were thoroughly examined for data extraction. The reference, study design, year of data collection, sample size, percentage of males, mean age, country, ethnic group, the gene that was relevant to our study, gene function, and implications on PD, variants, and the main conclusion of the included articles were extracted by several authors independently. The final manuscript was formed by the remaining authors in light of the extracted data and was reviewed by a separate author. Screening of the references of included articles was also conducted; however, no additional articles were identified to be included.

Results

Search Results

**Figure 1:**
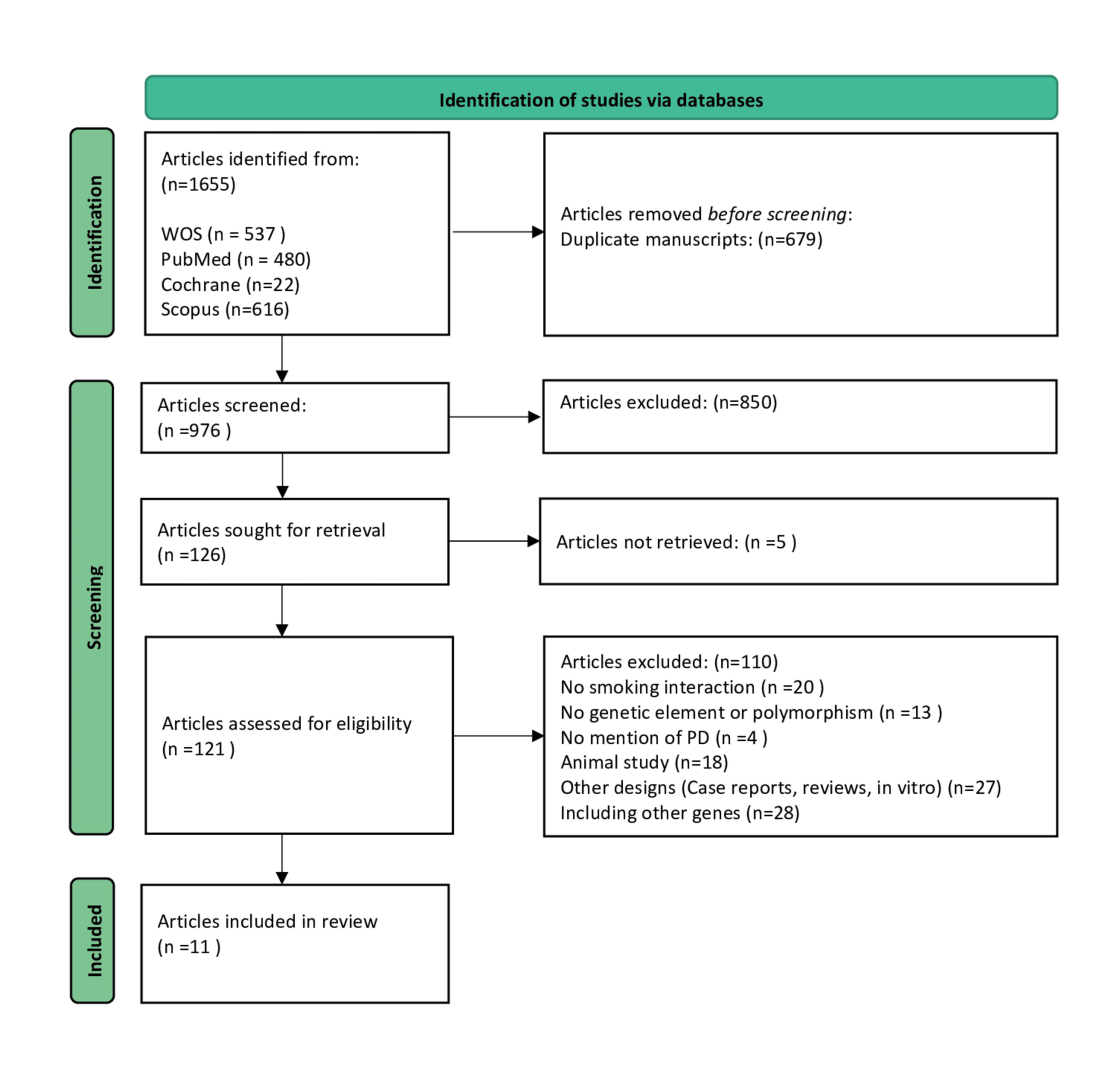
PRISMA flowchart of the review process PD: Parkinson’s disease; PRISMA: Preferred Reporting Items for Systematic reviews and Meta-Analyses; WOS: Web of Science

A comprehensive search was conducted across multiple databases, resulting in 1,655 articles. After removing duplicates, 976 articles were screened, 850 were excluded, and 126 were sought for retrieval. After eligibility evaluation, 110 articles were excluded, and 11 articles were included in the review (Figure 1) [13,22-29,34,35].

Characteristics of the Included Studies

The studies in this review, including a total of 5131 cases and 7542 controls, differ greatly in terms of study design, population demographics, and sample sizes. The participants were primarily diagnosed with PD, and studies included control groups for comparison analysis (Table 2). All studies included in this review were published more than ten years ago, specifically ranging from 2002 to 2011. The research covered adult populations with average ages ranging from 58 to 75 years. The majority of participants were male, with male percentages ranging from 54% to 100% among trials, with the majority of studies indicating a male predominance, typically around 60% or higher. The studies reviewed encompassed a diverse range of ethnic groups, primarily focusing on populations from North America, Japan, and Europe, with most tested being non-Hispanic whites (named in five studies).

**Table 2:** Summary of studies’ characteristics and findings COMT: catechol-O-methyltransferase; DRD: dopamine receptor D; MAO: monoamine oxidase; NR: not reported; PD: Parkinson’s disease; SV2C: synaptic vesicle glycoprotein 2C

Reference	Study design and study period	Number of cases/controls	Percentage of males (cases/controls)	Mean age (SD) (cases/controls)	Country/ethnic group	Genes	Smoking-PD relationship (OR for smoking)	Smoking-gene interaction on PD risk
Kiyohara et al. (2011) [13]	Case-control, 2006-2008	238/369	38.2/38.0	68.5/66.6	Japan	COMT, MAO-B, DRD2, and DRD4	Inverse relationship (OR = 0.56)	Evidence of interaction between COMT genotypes and smoking was suggested (p = 0.061). MAO-B genotypes showed a non-significant effect on the smoking-PD relationship. Polymorphisms in DRD2 and DRD4 did not affect the inverse relationship between smoking and PD.
Checkoway et al. (1998) [22]	Case-control, NR	98/146	58.1/60.9	69/71	USA/non-Hispanic whites	MAO-B	Inverse relationship (OR = 0.46)	Reduced PD risk related to smoking varied with the allelic distribution of the MAO-B gene.
Costa-Mallen et al. (2000) [23]	Case-control, NR	152/231	60.5/64.5	NR	USA/non-Hispanic whites	MAO-B and DRD2	Inverse relationship (OR = 0.54)	No statistically significant gene-tobacco interaction was observed for MAO-B or DRD2 polymorphisms in relation to PD.
Costa-Mallen et al. (2000) [24]	Case-control, NR	145/226	61.3/64.1	NR	USA/non-Hispanic whites	MAO-A and MAO-B	Inverse relationship	Relative risk trends for smoking-PD did not differ between MAO-A genotypes. A highly significant interaction was observed between smoking and MAO-B genotype.
De Palma et al. (2010) [25]	Case-control, NR	767/1989	56/53	69.8 (9.2)/69.8 (10.0)	Multicenter (Europe)/European origin population	MAO-B and DRD2	Inverse relationship (OR = 0.58)	No statistically significant gene-tobacco interaction was observed for MAO-B or DRD2 polymorphisms in relation to PD.
Gu et al. (2010) [26]	Case-control, 2000-2002	176/354	65.3/53.1	58.4 (10.1)/70.2 (7.0)	China/Han Chinese	SLC6A3, MAO-B, and COMT	Inverse relationship (OR = 0.14)	Genetic polymorphisms of SLC6A3, MAO-B, and COMT did not interact with the effect of smoking on reducing PD risk.
Hill-Burns et al. (2013) [27]	Case-control, NR	1600/1506	34.8/19.5	50/50	USA/European origin population	SV2C	Inverse relationship (OR = 0.81)	The effect of smoking on PD varied by SV2C genotype, ranging from protective to neutral to harmful.
Hernán et al. (2002) [28]	Case-control, 1976-1996	214/449	60.3/45.2	NR	USA/whites	MAO-B and COMT	Inverse relationship	The effect of smoking on PD did not change significantly with MAO-B or COMT genotypes.
Kelada et al. (2002) [29]	Case-control, 1993-2000	186/296	60.8/65.2	67.9/69.1	USA/non-Hispanic whites	MAO-A, MAO-B, and DRD2	Inverse relationship (OR = 0.60)	No significant overall or gender-specific interactions were found between smoking and MAO-A or DRD2 polymorphisms. However, a strong gender difference was observed in the modifying effect of the MAO-B genotype on the inverse smoking-PD relationship.
Tan et al. (2003) [34]	Case-control, NR	230/241	63/58.9	66 (9.2)/64 (9.4)	Singapore	MAO-B	Inverse relationship (OR = 0.57)	MAO-B genetic polymorphisms did not interact with the effect of smoking on reducing PD risk.
McGuire et al. (2011) [35]	Case-control, 1965-2005	1325/1735	61.1/65.4	64.9/68.4	North America/non-Hispanic whites (76%)	DRD2 and DRD3	Inverse relationship (OR = 0.7)	The inverse relationship between PD and smoking was not affected or modified by DRD2 or DRD3 genetic polymorphisms.

Smoking Relationship With PD

Across all studies, smoking was consistently associated with a reduced risk of PD [13,22-29,34,35]. This inverse relationship was noted regardless of the genetic background in all studies, supporting the hypothesis that smoking reduces the risk of PD. For instance, McGuire et al. (2011) found an inverse association between smoking and PD among non-Hispanic whites, with ORs (0.7) indicating a notable protective effect [35]. Similarly, Hill-Burns et al. revealed an OR of 0.81 of PD risk in smokers compared to nonsmokers in the European origin population [27]. Nevertheless, some authors suggested that the protective effect of smoking could be influenced by specific genetic polymorphisms, leading to variations in PD risk across different ethnic groups [22-24,27,29].

Gene-Smoking Relationship Impact on PD Across the Studies

**Figure 2:**
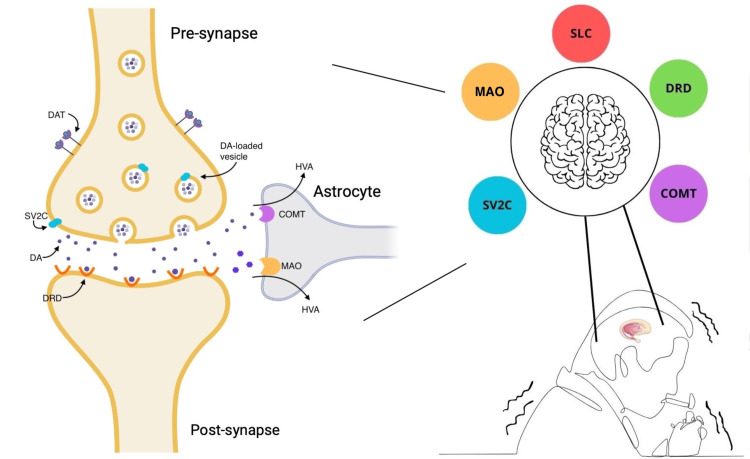
Simplified diagram illustrating the role of the evaluated genes in the review COMT: catechol-o-methyltransferase; DA: dopamine; DAT: dopamine transporter; DRD: dopamine D receptor; HVA: homovanillic acid; MAO: monoamine oxidase; SLC: solute carrier transport; SV2C: synaptic vesicle glycoprotein 2C [36-39]

The genes evaluated in the included studies included DRDs, MAO-A/B, COMT, SV2C, and solute carrier transport 6A3 (SLC6A3) genes, each with a detrimental role in the generation, transport, or metabolism of dopamine in the brain (Figure 2, Table 3) [36-39].

**Table 3:** Summary of dopamine pathway gene involvement in studies COMT: catechol-O-methyltransferase; DAT: dopamine transporter; DRD: dopamine receptor D; MAO: monoamine oxidase; mRNA: messenger ribonucleic acid; PD: Parkinson’s disease; SLC: solute carrier; SV2C: synaptic vesicle protein 2C; VNTR: variable number of tandem repeats

Gene	Function of the gene	Variants that were studied	Number of studies tested on	Conclusions on the effect on the smoking-PD relationship
MAO-A	MAO-A is implicated in the metabolism of neurotransmitters, including dopamine. Some studies have suggested MAO-A inhibition with smoking [24,29].	EcoRV (Y or N allele)	Two [24,29]	No significant interaction.
MAO-B	MAO-B is an essential enzyme in dopamine metabolism within the substantia nigra. It catalyzes the production of hydrogen peroxide and activates MPTP to MPP+, a toxic metabolite that can contribute to PD pathophysiology [13,34].	rs1799836 (Intron 13 G/A)	Nine [13,22-26,28,29,34]	In Japanese populations, smokers with the MAO-B genotype AA showed a nonsignificant increase in PD risk (OR = 2.39) compared with smokers with GA or GG combined. No significant interaction between smoking and MAO-B polymorphism was observed [13]. In non-Hispanic whites, a strong gender difference was found in the modifying effect of the MAO-B intron 13 genotype on the inverse smoking-PD relationship. Among ever-smoker men with the G genotype, PD odds were lower compared with never-smokers (OR = 0.27), while nonsmokers with the A genotype had higher PD risk (OR = 1.26). No interaction was detected in women [29]. Another study in non-Hispanic whites reported a highly significant interaction between smoking and MAO-B genotype, with smoking being protective for carriers of at least one G allele and showing no protective effect for individuals with only A alleles [24]. Similarly, in another study, the inverse smoking-PD relationship was confined to the G allele, while the A allele was associated with higher PD risk among smokers [22]. The total number of patients and controls with significant findings represented 16.5% and 13.14% of the total, respectively. Other studies reported insignificant results.
COMT	COMT is an enzyme that inactivates neurotransmitters and toxic catecholamines by methylation. Reduced COMT activity may lead to increased dopamine metabolism to neuromelanin, enhancing cytotoxic radical formation and contributing to neuronal degeneration [13].	Val108/158Met (rs4680)	Three [13,26,28]	Only one study in a Japanese population (238 (37.9%) cases and 369 (31.5%) controls) suggested evidence of interaction between COMT rs4680 genotypes and smoking (p = 0.061), with nonsmokers carrying at least one A allele having higher PD risk compared to ever-smokers with the GG genotype [13].
DRD2	DRD2 regulates, forms, and stores dopamine. In its polymorphisms, variants of the DRD2 gene show a C-to-T substitution, leading to mRNA instability and reduced translation, thereby causing reduced DRD2 expression in the cortex and striatum [35].	Taq1A (rs1800497) / Taq1B (rs1079597) / rs1076563 / rs6279 / rs6278 / rs273482 / rs1799732	Five [13,23,25,29,35]	No significant overall or gender-specific interactions of smoking with the Taq1A or Taq1B polymorphisms of DRD2 on PD.
DRD3	DRD3 regulates inflammatory mediators in astrocytes and microglia; DRD3 deficiency results in unresponsive astrocytes and attenuated microglial activation upon systemic inflammation [35].	rs6280 / rs2134655	One [35]	No significant interaction.
DRD4	DRD4 governs signaling effects and modulates motor behavior and activity of nigrostriatal neurons. Polymorphisms may influence susceptibility to PD [13].	rs1800955	One [13]	No significant interaction.
SLC	The SLC6 family of the human genome comprises transporters for neurotransmitters, amino acids, osmolytes, and energy metabolites. SLC6A2/NET and SLC6A3/DAT are implicated in dopamine transport [26].	VNTR (DAT)	One [26]	No significant interaction.
SV2C	Synaptic vesicle protein 2C is an integral membrane component of synaptic vesicles and has been implicated in the storage and release of neurotransmitters (dopamine). It is densely expressed in dopaminergic neurons in the substantia nigra, and changes in its expression may lead to altered synaptic transmitter release in PD pathogenesis [27].	rs30196 / rs10214163 / rs30196 + rs10214163	One [27]	The effect of smoking on PD varied by SV2C genotype, ranging from protective to neutral to harmful. The protective effect was strongest in individuals homozygous for the common alleles and decreased with the increasing number of minor alleles. Notably, no evidence was found for the association of smoking with the rs30196 genotype, the rs10214163 genotype, or the combined genotype of rs30196 and rs10214163. Therefore, the signal for the SV2C-smoking interaction on PD risk cannot be attributed to an association between the gene and smoking status.

DRD2-4 Genes

Five studies, with 2668 cases and 4620 controls, examined polymorphisms in the dopamine D2 receptor (DRD2) gene, with Taq1A (rs1800497) and Taq1B (rs1079597) being the commonest to be tested, and consistently found no modulation of DRD2 polymorphisms on the smoking effect on PD risk across diverse ethnicities and genders [13,23,25,29,35]. Regarding dopamine D3 and 4 receptors (DRD3 and 4), DRD3 polymorphism was evaluated in a case-control study of 1325 patients and 1735 controls, most of whom were non-Hispanic whites, and showed no effect on the smoking-PD relationship [35]. DRD4 was tested in only one study on 607 Japanese participants without any association with smoking-PD risk [13].

MAO-A and B Genes

The genetic polymorphism of MAO-A EcoRV (Y or N allele) was evaluated in two studies on non-Hispanic whites with a total of 331 cases and 522 controls and failed to show any difference in the smoking-PD relationship due to such polymorphism [24,29]. For instance, Costa-Mallen et al. (2000) showed no significant link between the polymorphism and PD, and the Y allele, associated with higher MAO-A enzyme activity, did not alter smoking’s protective effect against PD risk. Although there was a potential protective role in women, it was not statistically significant [24].

As for MAO-B genotypes, nine studies, with 2206 cases and 4301 controls, examined the relationship between smoking, the MAO-B intron 13 (rs1799836) genotype, and the risk of developing PD [13,22-26,28,29,34]. While most studies showed reduced risks of PD with smoking regardless of G/A polymorphism of MAO-B intro 13 (no modulation of genetics on smoking effect), only three studies on non-Hispanic whites, with 429 cases and 668 controls, showed significant differences. Costa-Mallen et al. (2000) revealed a protective effect of smoking in patients with the G allele, while A showed no protection [24]. Similarly, Checkoway et al. (1998) showed protection with the G allele and increased PD risks with the A allele [22]. Kelada et al. (2002), although a similar direction of effect was noted, the difference was confined to males only; males with the G genotype of MAO-B showed a protective effect from smoking, while those with the A genotype had an increased risk. No significant protective effect was observed in women [29]. Kiyohara et al. (2011) in their study on the Japanese population showed that allele A was associated with a non-significant increase in PD risks [13]. Overall, the significant effect of genotypes on the smoking-PD relationship was demonstrated in 365 PD cases and 565 controls, weighing around 16.5% and 13.14% of the total investigated cases and controls with MAO-B intron 13, respectively.

COMT Gene

The COMT gene polymorphisms were examined in three studies, with a total of 628 cases and 1172 controls, of mixed ethnicities: Japanese, Han Chinese, and European origin populations [13,26,28]. The study by Kiyohara et al. (2011), on 238 (37.9%) cases and 369 (31.5%) controls, found that individuals with the AA genotype of COMT rs4680 had a marginally non-significantly increased risk of PD compared to those with at least one G allele. The study also suggested an interaction between COMT genotypes and smoking status (p-value = 0.061) [13]. On the other side, Hernán et al. (2002) found no significant increase in PD risk associated with the COMT codon 158 polymorphism in individuals with heterozygous (HL) or homozygous (LL) COMT genotypes compared to those with high-activity homozygous (HH) genotypes. The frequency of COMT alleles was similar among cases and control subjects, indicating that the COMT gene may not play a significant role in PD susceptibility [28]. The study by Gu et al. (2010) found no significant association between COMT genotypes and PD risk in a Chinese population, with G/A and A/A genotypes. Smoking was inversely associated with PD risk, with the protective effect remaining consistent regardless of the COMT genotype [26].

SV2C and SLC6 Genes

Hill-Burns et al. (2013), in their investigation of 1600/1506 cases/controls of European origin, found that the effect of smoking on PD risk varied significantly with different SV2C (rs30196 and rs10214163) genotypes, demonstrating a protective, neutral, or harmful impact depending on the specific genetic background of individuals. Specifically, those homozygous for the common alleles exhibited a 56% risk reduction for PD associated with smoking, while individuals homozygous for the minor alleles showed a 223% risk increase [27]. On the other hand, Gu et al. (2011) in their study of 176/354 Han Chinese cases/controls revealed no modulating effect of SLC6A3 variants on the smoking-PD inverse relationship [26].

Discussion

Smoking has been consistently shown to have an inverse relationship with PD, potentially due to nicotine’s neuroprotective effects. However, the specific mechanisms and genetic factors involved in this protective effect remain unclear, and this protective effect is not uniform across all genetic backgrounds, indicating a gene-environment interaction [15,22,24-26,35]. The present scoping review, the first to investigate such a point, provides a thorough review of 11 case-control studies about the interaction between genetic polymorphism or variants of genes and smoking and its effects on PD risks.

Across studies, a consistent inverse relationship between smoking and PD risk was observed [13,22-29,34,35]. Focusing on genetic polymorphisms implicated in the dopaminergic pathway, which is central to PD pathology, no significant effect of DRD3, DRD4, SLC, or MAO-A polymorphisms on the inverse smoking-PD risk was noted. However, some studies identified specific genotypes of other genes that were associated with altered smoking-PD risks, and ethnicity and gender may have a crucial role [13,23,24,27,29].

MAO-B is an enzyme encoded by the MAO-B gene, located on the outer mitochondrial membrane. It plays a crucial role in the catabolism of neuroactive and vasoactive amines in both the central nervous system and peripheral tissues [6,11,22]. PD is associated with elevated MAO-B levels in the brain and the production of the toxic MPP+. Thus, reduced MAO-B activity may confer a protective effect against PD risk. It has been reported that cigarette smoking suppresses MAO-B gene activity, highly suggesting an interaction on the PD risk [13,34]. The A/G allele distribution has been linked to differences in enzymatic activities. While most studies failed to show the effect of MAO-A or MAO-B polymorphism on smoking effect [23,25,26,28,34], two studies showed that the protective smoking was only in those with the G allele [22,24]. Gender-based difference was detected where the G allele was protective in men, while women showed no change according to allele distribution [29]. It is noteworthy that the described changes in PD risks with different genotypes were demonstrated in only 16.5% and 13.14% of the total investigated cases and controls, and thus, such findings cannot be generalized without further extensive investigations.

The COMT enzyme inactivates dopamine, levodopa, and other neurotoxic catechols through methylation. Reduced COMT activity has been associated with increased dopamine breakdown into neuromelanin, potentially enhancing cytotoxic radical formation and contributing to neuronal degeneration [28,40]. The A allele of COMT rs4680 is linked to decreased soluble COMT activity and may elevate PD risk, as suggested by Kiyohara et al. (2011), particularly in nonsmokers with at least one A allele [13]. Other studies supported an inverse association between smoking and PD across all investigated COMT genotypes [13,26].

The SV2C gene, expressing SV2C, due to its role in enhancing dopamine release via synaptic vesicles, can be a credible candidate for PD investigations, although its polymorphisms have been studied with smoking in only one study [26,41]. The study reported mixed findings, with effects ranging from neutral to potentially adverse in the context of the smoking-PD association, depending on the specific SV2C genotype, suggesting that smoking’s or nicotine’s efficacy as a neuroprotective treatment may differ among individuals, so only a subset of the population will benefit from nicotine as a protective measure against PD [26].

Limitations

Limited studies are available on the gene-smoking interaction in PD, with some genes studied in only one or a few studies, and the available studies were conducted on particular ethnic groups, with most of the population being non-Hispanic whites. The retrospective nature of the studies may introduce bias. The inconsistent results for some genes may be due to the multifactorial nature of PD, as well as the influence of other previously reported gene polymorphisms that may interfere with our study.

Recommendations

Given the potential protective effects of smoking, especially with certain genotypes, clinicians might consider the use of nicotine or related compounds as part of a treatment regimen for patients whose genotypes show protective effects with smoking (e.g., common alleles of the SV2C gene). However, this would require careful consideration of the individual’s genetic profile and potential risks. Genetic counseling for PD could be enhanced by incorporating information about the specific polymorphisms discussed in the review. Genetic screening could become a part of routine assessments for individuals at risk of PD, allowing for customized interventions based on their genetic profile and lifestyle factors, such as smoking history. For individuals at higher genetic risk, genetic counselling can include discussions on lifestyle choices, including the potential benefits and risks of smoking or nicotine use, tailored to their genetic profile. Since gender differences in gene-smoking interactions were noted on some occasions, clinicians might need to consider gender-specific strategies when advising patients on PD risk and management.

As for future research, due to the limited studies, more studies representing different ethnicities, genders, and genetic backgrounds would provide a comprehensive evaluation. Furthermore, future clinical trials could stratify participants based on genotypes with smoking interaction to evaluate treatment responses and optimize therapeutic approaches. Biomarkers with prognostic indicators, such as MAO-B and COMT activity levels, influenced by genetic variations, could serve as biomarkers to predict PD progression or the effectiveness of neuroprotective interventions.

## Conclusions

The present review extends the inverse relationship between smoking and PD, although the underlying genetic and mechanistic basis of this effect remains elusive. Of the investigated genes in the dopaminergic pathway, changes in the smoking-PD relationship were noted with polymorphisms in the MAO-B, COMT, and SV2C genes, highlighting the potential gene-smoking interaction. Although such findings pave the way for treatment or prevention strategies, further large-scale research with proper representation of ethnicities, gender, and genes is needed to validate such results.
